# Features of Age-Related Macular Degeneration in the General Adults and Their Dependency on Age, Sex, and Smoking: Results from the German KORA Study

**DOI:** 10.1371/journal.pone.0167181

**Published:** 2016-11-28

**Authors:** Caroline Brandl, Valentin Breinlich, Klaus J. Stark, Sabrina Enzinger, Matthias Aßenmacher, Matthias Olden, Felix Grassmann, Jochen Graw, Margit Heier, Annette Peters, Horst Helbig, Helmut Küchenhoff, Bernhard H. F. Weber, Iris M. Heid

**Affiliations:** 1 Department of Genetic Epidemiology, University of Regensburg, Regensburg, Germany; 2 Department of Ophthalmology, University Hospital Regensburg, Regensburg, Germany; 3 Institute of Human Genetics, University of Regensburg, Regensburg, Germany; 4 Statistical Consulting Unit StaBLab, Department of Statistics, Ludwig-Maximilians-University Munich, Germany; 5 Institute of Developmental Genetics, Helmholtz-Zentrum München, Neuherberg, Germany; 6 Institute for Epidemiology II, Helmholtz-Zentrum München, Neuherberg, Germany; 7 Institute of Genetic Epidemiology, Helmholtz Zentrum München, German Research Center for Environmental Health, Neuherberg, Germany; University of Rochester Medical Center, UNITED STATES

## Abstract

Age-related macular degeneration (AMD) is a vision impairing disease of the central retina characterized by early and late forms in individuals older than 50 years of age. However, there is little knowledge to what extent also younger adults are affected. We have thus set out to estimate the prevalence of early AMD features and late AMD in a general adult population by acquiring color fundus images in 2,840 individuals aged 25 to 74 years of the Cooperative Health Research in the Region of Augsburg project (KORA) in South Germany. Among the 2,546 participants with gradable images for each eye, 10.9% (n = 277) had early AMD features (applying the 9-step Age-Related Eye Disease Study Severity Scale), 0.2% (n = 6) had late AMD. Prevalence increased with age, reaching 26.3% for early AMD features and 1.9% for late AMD at the age 70+. However, signs of early AMD were found in subjects as young as 25 years, with the risk for early AMD features increasing linearly by years of age in men, and, less consistent with a linear increase, in women. Risk for early AMD features increased linearly by pack years of smoking in men, not in women, nor was there any association with other lifestyle or metabolic factors. By providing much sought-after prevalence estimates for AMD from Central Europe, our data underscores a substantial proportion of the adult population with signs of early AMD, including individuals younger than 50 years. This supports the notion that early AMD features in the young might be under-acknowledged.

## Introduction

Age-related macular degeneration (AMD) is a degenerative disorder of the central retina and represents the leading cause of severe visual impairment in the elderly population of industrialised societies.[1–3] Deteriorative disturbances in the functional syncytium of choriocapillaris, retinal pigment epithelium (RPE) and neurosensory retina finally result in loss of central vision through the death of photoreceptors.[4]

This clinical endpoint is also termed late AMD, which can appear as a wet form characterised by choroidal/sub-retinal neovascularisation (NV), or a dry form, known as geographic atrophy (GA) of the RPE.[2, 3] Therapeutic options for late AMD are limited: clinically effective treatments are currently restricted to anti-VEGF therapy for wet late AMD; the treatment is no cure, but rather slows down the progression of vision loss.[5–7]

Late AMD is typically preceded by early AMD stages, which are usually clinically asymptomatic and therefore largely remain unacknowledged. Early AMD features are determined by differently sized yellowish accumulations of extracellular material (drusen) that are deposited between Bruch's membrane and the RPE. Other signs of early AMD are RPE abnormalities, including depigmentation or increased amount of pigment.[2–4]

Multiple factors are known to play a role in the pathophysiology of AMD. The strongest risk factor is age: late AMD can be found primarily in those aged 70 years and older.[2, 3] Cigarette smoking has also been reported consistently to be associated with late and early AMD.[8–14] Weaker and contradicting findings of association with AMD were reported for other lifestyle factors such as dietary behaviour or physical activity and for metabolic parameters like hypertension, diabetes mellitus, body mass index, HDL/LDL cholesterol and triglyceride levels, among others.[8–10, 15–19] Unresolved is the earlier claim of potentially higher AMD prevalence among women.[3, 13, 20] Moreover, AMD is influenced by genetic risk variants.[21]

Early AMD stages are highly relevant as individuals with early AMD are at increased risk of progressing to late the stage form.[8, 22] Among the subjects with more severe stages of early AMD, like large drusen or pigmentary changes present in both eyes, 50% of these are predicted to progress to late AMD within 5 years.[23, 24] Due to the still unsatisfying therapy options for late AMD, subjects with early AMD comprise an ideal target group for preventive measures–if just such measures were available.[24]

Early and late AMD are generally defined for subjects who exhibit the above mentioned features on color fundus images of the central retina and who are at least 50 years of age.[25] There is little knowledge to what extent younger adults carry features of early AMD or even late AMD. This is due to the fact that the clinical presentation is usually by the elderly and that epidemiological studies on AMD have usually limited their investigation to those older than 40 or 50 years.[26]

In the present study, we have thus set out to determine the prevalence of features of early and late AMD in a population-based study of the general adults covering those at 25 to 74 years of age from a midsized city in South Germany. We moreover aimed to describe the dependency on age, sex, lifestyle factors, and metabolic parameters. This data will also add to a gap of data from Germany and Central Europe.

## Subjects and Methods

### Study population and study sample

The Cooperative Health Research in the Region of Augsburg project (Kooperative Gesundheitsforschung in der Region Augsburg, KORA) is a research platform to survey the development and course of chronic diseases.[27] Details have been described previously.[27, 28] In brief, four cross-sectional health surveys were performed among the inhabitants of Augsburg, a middle sized city in the South of Germany, and surrounding counties. The present analysis is based on the fourth KORA survey (KORA-S4), conducted 1999–2001. Subjects were included if they had a German passport, had their primary residence in the study region, were 25–74 years of age, and were able and willing to provide written informed consent–yielding 4,261 participants. This survey exhibited a high response of 67% [29] and is thus reasonably representative. Of the full survey, 2,840 participants were enrolled in the fundus sub-study, which were all participants in one of the recruiting centers being equipped with a color fundus camera.

The Ethics Committee of the Bavarian Medical Association (Bayerische Landesärztekammer) and the Bavarian commissioner for data protection and privacy (Bayerischer Datenschutzbeauftragter) approved the study, which complies with the 1964 Helsinki declaration and its later amendments. Informed written consent was obtained from all individual participants included in the study.

### Assessment of lifestyle factors and metabolic parameters

Information on lifestyle factors including smoking, physical activity and healthy diet were gathered by trained medical staff during a standardised face-to-face interview. Smoking was quantified as pack years, calculated by multiplying the number of packs of cigarettes smoked per day by the number of years the person has smoked. Participants were classified as current smokers (including regular smokers, currently smoking ≥ 1 cigarette per day, and occasional smokers, currently smoking < 1 cigarette per day), ex-smokers, and never smokers. Physical activity was assessed as regular activity during leisure time in summer and winter weekly for ≥ 1 hour (active) or less (not active).[30] A score for healthy diet was computed as described previously [31] based on a 24-item standardized questionnaire and dichotomized at the median of the study sample (healthy diet: score ≥ 15.00, no healthy diet: score < 15.00).

The metabolic parameters body-mass-index (BMI), type 2 diabetes mellitus (T2DM), hypertension, and cholesterol levels were assessed by physical examination, interview information, and laboratory measurements: BMI (kg m^-2^) was computed based on measured weight in kg (in light clothing, to nearest 0.1 kg) and height in m (to nearest 0.5 cm) as weight divided by squared body height.[32] T2DM was assessed as self-reported type 2 diabetes or reported anti-diabetes therapy intake. Hypertension was assessed as actually measured systolic blood pressure of ≥ 140 mmHg, diastolic blood pressure of ≥ 90 mmHg or corresponding medication taken, given that the participants were aware of having hypertension High and low density lipoprotein cholesterol (HDL-C, LDL-C) was measured as described previously.[28]

### Acquisition and grading of color fundus images of the central retina

The KORA-S4 survey was conducted in several study centers, one of them being equipped for color fundus imaging. Therefore, 2,842 of the 4,261 KORA-S4 participants were eligible for the fundus sub-study, of which 2,840 agreed to the fundus photography and were thus enrolled into the fundus sub-study. At least one non-stereoscopic color fundus photograph of the central retina of each eye, including full macular region and optic disc, was acquired using a 45° non-mydriatic fundus camera (TRC-NW5S, Topcon, Willich, Germany). Images were available as.jpg-files with a resolution of 768 x 576 pixels.

Acquired photographs were defined as gradable if fulfilling certain quality criteria allowing for the precise assessment of AMD-related abnormalities. Quality criteria included image brightness, color contrast, full macular region captured on the images. Subjects were excluded, if no fundus images were available for any eye, fundus images were ungradable for either eye, fundus images of at least one eye showed obscuring lesions (e.g. cataract) or lesions that were considered to be the result of a competing eye/retinal disease (such as diabetic retinopathy, high myopia, trauma, congenital diseases, or photocoagulation unrelated to choroidal neovascularization). If more than one image per eye was available and gradable, only the best image with regard to centering and quality was selected for further assessment, i.e. only one image per eye was evaluated as follows.

Gradable images were examined by one trained grader (V.B.) who was blinded towards age, sex, and other information on the participant. Questionable findings were discussed with a senior grader (C.B., ophthalmological consultant). Images were evaluated applying a standard digital grid template adapted from the Early Treatment Diabetic Retinopathy Study (ETDRS) that was placed over each image, adjusting for size, using Gimp (GNU Image Manipulation Program, version 2.6.11) as shown in **S1 Fig**. Only lesions within the grid were considered. For each eye, drusen area and presence of pigment abnormalities (including hyperpigmentation, depigmentation and paracentral geographic atrophy) were assessed. Early AMD stages were graded following the 9-step Age-Related Eye Disease Study (AREDS) Severity Scale from AREDS report no. 17. [33], which combines a 6-step drusen area scale with a 5-step pigmentary abnormality scale. Central geographic atrophy (GA) and choroidal/sub-retinal neovascularisation (NV) with scaring or haemorrhage were assessed as late AMD.[2, 3] GA was defined as GA only if no NV was present; NV was defined if NV was present no matter whether additionally GA was present in the same or the other eye. A summary of the grading criteria is given in **S1 Table**. Persons were classified based on the eye with the more severe grade. Only participants with at least one gradable image for each eye were included in further analyses.

### Statistical analysis

Prevalence estimates were derived from the cross-sectional data using the Bavarian population for standardization (www.statistikdaten.bayern.de/genesis/). The association of early AMD with age, sex, smoking, other lifestyle factors or metabolic parameters was presented as odds ratio per unit change in the respective covariate.

To evaluate the trend of AMD risk by year of age or by pack year, we applied generalized linear models with logistic link and thin plate regression splines [34, 35]: We explored the degrees of freedom (df) for evidence of linearity (df ~ 1) and adopted a linear model where appropriate. The unbiased risk estimation score (UBRE-score) with a modification from Kim and Gu, 2004 [36] was used to estimate the smoothing parameters. We accounted for potential interaction of age or pack year with sex.

All analyses were carried out using statistical software packages SPSS or “R” (Version 3.1.1, The R Foundation for Statistical Computing) and the package mgcv.[34, 37]

## Results

### Analysed study sample and subject characteristics

Of the 2,840 KORA-S4 participants enrolled into the fundus sub-study, central fundus photographs were successfully acquired for each eye from 2,751 individuals (96.9%). Of these, 2,546 participants had a gradable image for each eye without competing retinal disease (92.5% compared to those with acquired photographs, **S2 Table**). About half of the analysed subjects were women (49.8%); age at examination ranged from 24 to 75 years. Almost a third of the subjects were current smokers (29.0%), another third ex-smokers (31.6%), 21.5% were obese (i.e. BMI ≥ 30 kg m^-^^2^), 3.5% had T2DM, and 28.3% had hypertension (**Table 1**).

**Table 1:** Characteristics of analysed subjects from the KORA-S4 fundus sub-study.

		All	Men	Women	n
	Age [years], mean ± SD	47.5 ± 13.6	48.0 ± 13.7	46.9 ± 13.5	2546
	Age ≥ 50 years, n (%)	1125 (44.19)	592 (46.32)	533 (42.03)	2546
**Lifestyle factors**					
	Pack years^**a**^, mean ± SD	11.2 ± 17.8	15.5 ± 21.6	6.8 ± 11.5	2499
	Current smoker^**b**^, n (%)	736 (28.95)	418 (32.78)	318 (25.10)	2542
	Ex-smoker, n (%)	803 (31.59)	473 (37.10)	330 (26.05)	2542
	Physically active^**c**^, n (%)	1291 (51.07)	635 (50.16)	656 (51.98)	2528
	Healthy diet^**d**^, n (%)	1412 (55.85)	613 (48.42)	799 (63.31)	2531
**Metabolic parameters**					
	BMI [kg m^-^^2^]^**e**^, mean ± SD	26.87 ± 4.73	27.29 ± 4.17	26.45 ± 5.21	2531
	T2DM^**f**^, n (%)	89 (3.50)	55 (4.32)	34 (2.68)	2542
	Hypertension^**g**^, n (%)	717 (28.33)	474 (37.32)	243 (19.27)	2531
	HDL-C [mg/dl], mean ± SD	57.62 ± 17.12	51.06 ± 13.72	64.23 ± 17.65	2532
	LDL-C [mg/dl], mean ± SD	136.07 ± 42.29	142.29 ± 41.49	129.81 ± 42.18	2529

Shown are subject characteristics for those with gradable fundus images for each eye and no competing retinal disease (n = 2546), separately for men (n = 1278) and women (n = 1268). Abbreviations: SD = standard deviation; BMI = body-mass-index; T2DM = type 2 diabetes; HDL-C, LDL-C = high and low density lipoprotein cholesterol

^**a**^) Pack years are defined as number of packs (20 cigarettes per pack) smoked per day times the number of years of smoking.

^**b**^) Current smokers are defined as regular smokers currently smoking ≥ 1 cigarette per day, and occasional smokers, currently smoking < 1 cigarette per day.

^**c**^) Physically active is defined as ≥ 1 hour of activity per week during leisure time in summer and winter.

^**d**^) Healthy diet is defined as a healthy diet score above the median of the analysed sample (median score = 15.00).

^**e**^) BMI is defined as measured weight divided by squared measured body height.

^**f**^) T2DM is defined as a self-reported diagnosis or anti-diabetes medication intake.

^**g**^) Hypertension is defined as actually measured systolic blood pressure of ≥ 140 mmHg, diastolic blood pressure of ≥ 90 mmHg or corresponding medication taken, given that the participants were aware of having hypertension.

Of note is a dependency of the smoking habit on sex and age. While the proportion of current smokers decreased by age for both men and women, the proportion of ever smokers (current or ex-smoker) was rather constant across age for men, but not for women: older women (≥ 60 years old, i.e. born before 1940) were less likely ever smokers compared to younger women or men (~30% for older, 50–60% for younger women, 60–70% for men, **S3 Table**). This is in line with persons tending to stop smoking at advanced age and/or a cohort effect of a time when smoking was not yet fashionable for women.

### Comparison of the analysed study sample with the full KORA-S4 survey

To evaluate whether there were general differences between the subjects in our fundus sub-study compared to the full KORA-S4 survey including 4,261 participants, we compared study characteristics for subjects for whom fundus images were not obtained (n = 1,472), for those with acquired, but ungradable images or competing retinal disease (n = 243), or those with gradable images for each eye without competing retinal disease (analysed subjects, n = 2,546). Analysed participants were significantly younger than those without imaging or those without grading (mean age = 47.5 versus 50.2 or 60.5 years, P for difference = 7.56*10^−10^ and 9.80*10^−46^, respectively, **S4 Table**, **S2 Fig**). To some extent, this may be due to diminished compliance of elderly to fundus exam or/and a smaller pupil size in the elderly prohibiting non-mydriatic fundus photography with sufficient quality.

Consistent with the distribution of the analysed subjects shifted towards the younger, the distributions of all age-dependent metabolic parameters were shifted towards lower BMI, less T2DM, less hypertension, and lower levels of LDL-C (**S4 Table)**. There were more current smokers among the analysed subjects compared to the un-imaged or un-gradable subjects (28.9% versus 22.1% or 18.9%, respectively), but the dependency of smoking on sex and age in the full KORA cohort was similar compared to the analysed subjects (data not shown).

### Observed frequency and estimated prevalence of early and late AMD

Among the 2,546 analysed participants, early AMD features (AREDS severity steps 2 to 9) were observed for 10.9% of the subjects consisting of 8.0% (n = 204) with AREDS severity steps 2 or 3 and 2.9% (n = 73) with AREDS severity steps 4 to 9, the latter demonstrating more distinct early AMD pathologies. Using the Bavarian population as a reference, age- and sex-standardized prevalence for early AMD was estimated as 11.4% (**Table 2**). When separating by sex, standardized prevalence of any early AMD features was 10.6% for men and 12.1% for women. When limiting to subjects with ≥ 40 years of age, standardized prevalence of any early AMD features was 13.8% for overall, 13.5% for men and 14.1% for women (**Table 2**). Applying a Mantel-Haenszel Test controlling for ten-year age-groups, we found no significant difference between the prevalence of women compared to men (P = 0.372 overall, P = 0.976 restricting to those ≥ 40 years of age).

**Table 2:** Observed frequency and prevalence of early/late AMD and its dependency on age and sex.

Age groups (years)	<30	30–39	40–49	50–59	60–69	70–75	All	Prevalence^a^	
								All	≥40
N in study	229	644	548	517	452	156	2546	-	-
N men	109	311	266	273	232	87	1278	-	-
N women	120	333	282	244	220	69	1268	-	-
N in Bavarian population	769000	1557222	2128383	1729919	1349267	709133	8242924	-	-
N men	386600	782873	1084206	865646	658797	329606	4107728	-	-
N women (weight^a^)	382400	774349	1044177	864273	690470	379527	4135196	-	-
**Overall**									
Early AMD^b^, % (n)	3.06 (7)	6.37 (41)	10.04 (55)	10.64 (55)	17.26 (78)	26.28 (41)	10.88 (277)	11.35	13.78
AREDS steps 2+3, % (n)	2.62 (6)	5.59 (36)	9.12 (50)	7.93 (41)	10.62 (48)	14.74 (23)	8.01 (204)	8.29	9.77
AREDS steps 4+, % (n)	0.44 (1)	0.78 (5)	0.91 (5)	2.71 (14)	6.64 (30)	11.54 (18)	2.87 (73)	2.86	3.98
Late AMD (GA and/or NV), % (n)	0.00 (0)	0.16 (1)	0.00 (0)	0.00 (0)	0.44 (2)	1.92 (3)	0.24 (6)	0.23	0.29
**Men**									
Early AMD^b^, % (n)	0.92 (1)	4.50 (14)	7.89 (21)	13.19 (36)	17.67 (41)	24.14 (21)	10.49 (134)	10.58	13.47
AREDS steps 2+3, % (n)	0.92 (1)	3.86 (12)	6.77 (18)	8.79 (24)	9.91 (23)	13.79 (12)	7.04 (90)	7.04	8.97
AREDS steps 4+, % (n)	0.00 (0)	0.64 (2)	1.13 (3)	4.40 (12)	7.76 (18)	10.34 (9)	3.44 (44)	3.44	4.90
Late AMD (GA and/or NV), % (n)	0.00 (0)	0.32 (1)	0.00 (0)	0.00 (0)	0.43 (1)	3.45 (3)	0.39 (5)	0.39	0.47
**Women**									
Early AMD^b^, % (n)	5.00 (6)	8.11 (27)	12.06 (34)	7.79 (19)	16.82 (37)	28.99 (20)	11.28 (143)	12.12	14.08
AREDS steps 2+3, % (n)	4.17 (5)	7.21 (24)	11.35 (32)	6.97 (17)	11.36 (25)	15.94 (11)	8.99 (114)	8.99	10.43
AREDS steps 4+, % (n)	0.83 (1)	0.90 (3)	0.71 (2)	0.82 (2)	5.45 (12)	13.04 (9)	2.29 (29)	2.29	3.07
Late AMD (GA and/or NV), % (n)	0.00 (0)	0.00 (0)	0.00 (0)	0.00 (0)	0.45 (1)	0.00 (0)	0.08 (1)	0.08	0.12

Shown are the proportions of subjects with early/late AMD in the 2,546 analysed subjects. Also given is the number of inhabitants of Bavaria in 2011, which were used to compute the age- and sex-standardized prevalence estimates. Abbreviations: AREDS = Age-Related Eye Disease Study; GA = geographic atrophy; NV = neovascularisation

^a^) Ten-year-age-group- and sex-standardized prevalence estimates based on the weights from the Bavarian population.

^b^) Early AMD is defined as AREDS severity steps 2–9.

The frequency of observed early AMD features increased by age (**Table 2, S3 Fig**): from 3.1% in young adults (< 30 years) to 26.3% in the elderly (70+ years). This corresponds to a 9-fold risk increase when comparing the elderly to young adults (**S5 Table**). The increased trend by age was also apparent when focusing on subjects graded as AREDS severity steps 4+ or steps 2+3 and we observed the more individuals with higher severity steps the higher the age (**Table 2, S6 Table**). However it should be noted that signs of early AMD were found for subjects as young as 28 or 25 years, respectively.

As expected for a general adult population, the number of subjects with late AMD was low: six subjects had late AMD (0.2%), one with GA and five with NV. We observed a tendency towards increased late AMD frequency by age from <0.1% for those aged ≤60 years to 0.4% and 1.9% for those at 60–69 and 70+ years, respectively (**Table 2**). Standardized late AMD prevalence was 0.2% overall (0.4% in men, 0.1% in women). However, the available number of subjects with late AMD in this study sample is too low for further conclusions. We therefore excluded subjects with late AMD for subsequent analysis.

While AMD features were assessed by person based on the eye with the more severe grade according to the guideline [33], we also compared features between the eyes for each person. We found 2,380 individuals (93.5% of the 2,546 analysed subjects) with the same AMD status in both eyes (**S7 Table**).

### Modelling the risk for early AMD features by age and sex

**Fig 1:**
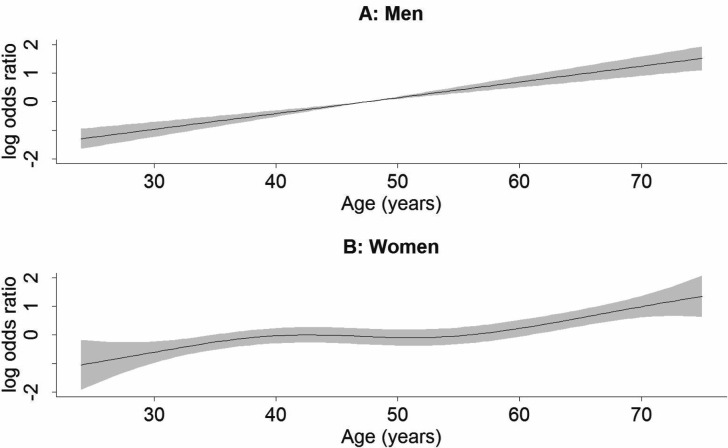
The age-trend of early AMD risk modelled separately for men and women. Shown is the modelled trend of early AMD relative risk by years of age utilizing a thin plate regression spline for **A)** men (n early AMD = 134, n AMD-free = 1,139) or **B)** women (n early AMD = 143, n AMD-free = 1,124). Relative risk is given as log odds ratio with persons at the age of 50 as reference and 95% confidence interval shaded in grey. The modelled trend was in line with a linear relationship for men (degrees of freedom = 1.001), but not for women (degrees of freedom = 3.283).

When modelling the risk for early AMD features (defined as AREDS severity steps 2–9) by age (approximated by odds ratios, OR, via logistic regression models) in order to describe the “dose-response” relationship accounting for a potentially different age-trend by sex, we obtained several interesting findings: (1) when modelling the age-effect linearly, we found a significant increase in the risk of early AMD features per year which differed between men and women (men: OR = 1.057 per year, P < 0.001; women: OR = 1.035, P < 0.001; P for difference = 0.034). (2) When allowing for a non-linear trend by year of age utilizing sex-specific thin plate regression spines, we found evidence for a non-linear relationship for women exhibiting a plateau across the age of 40 to 55 years, but a linear relationship for men (**Fig 1**, men: degrees of freedom (df) = 1.001; women: df = 3.283, df ~ 1 indicates a linear relationship). When restricting to those above 55 years, the age-trend by year was linear and similar for both women and men (men: df = 1.000 from spline model, OR = 1.066 from linear model; women: df = 1.000, OR = 1.082). (3) When separating the analysis for those with AREDS severity steps 4+ from those with AREDS steps 2+3, each compared to controls, we found that the plateau of steady early AMD risk in women is mostly due to AREDS step 2+3 features (women: df = 2.917 for AREDS steps 2+3, df = 1.975 for AREDS steps 4+; men: df = 1.003 for AREDS steps 2+3, df = 1.001 for AREDS steps 4+, **S4 Fig**).

Altogether, our data supports a linearly increased risk of early AMD features by years of age for men, but a plateau of steady early AMD risk for women in the period between 40 to 55 years of age.

### AMD association with smoking

Early AMD features are usually clinically asymptomatic, while late AMD is diagnosed when subjects experience first signs of visual impairment. In a cross-sectional survey, the association with lifestyle factors may thus be biased by late AMD patients changing lifestyle or reflecting their lifestyle differently upon diagnosis, while the association of early AMD with concurrently reported lifestyle may be considered to be genuine. Therefore, we analysed life-long smoking behaviour for association with early AMD.

First, when comparing ever-smoker (current or ex-smoker) to never-smoker with regard to their risk for early AMD features, we found no significant association and diverging estimates for men and women (P overall = 0.112; men: OR = 1.422, P = 0.107; women: OR = 0.790, P = 0.205; model accounting for interaction of smoking with sex and sex-specific spline for age, **Table 3**). However, we did observe a significant association in men, but not in women, when restricting to subjects graded as AREDS severity steps 4+ (P overall = 0.010; men: OR = 2.944, P = 0.017; women: OR = 0.623, P = 0.258). Similar results were found when modelling age linearly (**Table 3**).

**Table 3:** Association of early AMD features with smoking.

	Model	Overall^a^	OR men (95% CI)	P men	OR women (95% CI)	P women	P sex diff^b^
**Early AMD**^**c**^							
	Ever/never smoker						
	+ age non-linear	0.112	1.422 (0.927; 2.180)	0.107	0.790 (0.549; 1.137)	0.205	0.040
	+ age linear	0.109	1.422 (0.927; 2.180)	0.107	0.787 (0.549; 1.128)	0.192	0.038
	PY						
	+ age non-linear	0.059	1.007 (1.000; 1.014)	0.041	0.989 (0.972; 1.006)	0.201	0.051
	+ age linear	0.045	1.007 (1.000; 1.014)	0.041	0.988 (0.971; 1.005)	0.151	0.035
**AREDS steps 4+**							
	Ever/never smoker						
	+ age non-linear	0.010	2.944 (1.218; 7.115)	0.017	0.623 (0.274; 1.416)	0.258	0.012
	+ age linear	0.009	2.944 (1.218; 7.115)	0.017	0.604 (0.266, 1.370)	0.228	0.010
	PY						
	+ age non-linear	0.003	1.014 (1.005; 1.024)	0.003	0.951 (0.895; 1.010)	0.102	0.038
	+ age linear	0.002	1.014 (1.005; 1.024)	0.003	0.949 (0.893; 1.007)	0.084	0.031

Shown are odds ratios (OR) and P-values from logistic regression models with smoking as dichotomous (ever versus never smoker) or quantitative variable (pack year, PY, modelled linearly), presenting one model per row. The outcome is early AMD versus controls (for ever/never smoking (PY), 276 (272) early AMD subjects versus 2,260 (2,221) AMD-free) or AREDS steps 4+ versus controls (for ever/never smoking (PY), 73 (72) AREDS steps 4+ subjects versus 2,260 (2,275) AMD-free). All models include the covariates: smoking as noted in the table, sex (0 = men, 1 = women), age (in years), interaction of smoking with sex, and a sex-specific linear or non-linear (using a thin plate regression spline) trend by age. Abbreviations: PY = pack year; AREDS = Age-Related Eye Disease Study

^a^) Likelihood ratio test for testing the main effect smoking and the interaction sex x smoking simultaneously.

^b^) P-value for the interaction of the smoking variable and sex.

^c^) Early AMD is defined as AREDS severity steps 2–9.

Second, we analysed pack year as a cumulative measure for the amount smoked during a lifetime for association with early AMD features. We found no evidence for non-linearity (men: df = 1.003; women: df = 1.000; **S5 Fig,** model with sex-specific splines for pack year and age). We thus adopted a linear model for pack year and observed borderline significance for an increased risk of early AMD features by pack year in men, but not in women (P overall = 0.059, men: OR = 1.007 per pack year, P = 0.041; women: OR = 0.989, P = 0.201, model with interaction of pack year with sex and sex-specific spline for age, **Table 3**). As before, this association in men was more pronounced when restricting the analysis to subjects graded as AREDS severity steps 4+ (P overall = 0.003; men: OR = 1.014, P = 0.003; women: OR = 0.951, P = 0.102). The results were very similar when the age trend was modelled linearly (**Table 3**). It should be noted that our study included fewer elderly women who have ever smoked or currently smoked compared to men. In summary, we find an association of early AMD features with smoking, which increases by pack year, but only for men, not for women.

### Association of early AMD with other lifestyle or metabolic factors

To extend on lifestyle factors, we investigated the association of a healthy diet or a physically active lifestyle with risk for early AMD features using a model adjusted for age (linearly and non-linearly as sensitivity analysis), sex, pack year (linearly), and interaction effects sex x age and sex x pack year. We found no statistically significant association with healthy diet or physical activity, neither with any early AMD features nor when restricting to subjects graded as AREDS severity steps 4+ (**Table 4A**). Modelling of age non-linearly provided similar results (data not shown).

**Table 4:** Association of early AMD features with lifestyle or metabolic factors.

	Model	covariate	P^a^	OR (95% CI)
**A) Lifestyle**				
	Early AMD^b^	Age (year)	<0.001	m: 1.056 (1.040; 1.072); w: 1.032 (1.018; 1.046)
		Sex (0 = men, 1 = women)	0.007	1.518 (1.102; 2.093)
		Smoking (pack year)	0.033	m: 1.008 (1.001; 1.015); w: 0.987 (0.971; 1.004)
		Healthy diet (1 = yes, 0 = no)	0.441	1.115 (0.845; 1.472)
		Physically active (1 = yes,0 = no)	0.825	1.030 (0.791; 1.342)
	AREDS steps 4+	Age (years)	<0.001	m: 1.093 (1.059; 1.128); w: 1.084 (1.048; 1.121)
		Sex	0.011	1.270 (0.605; 2.669)
		Smoking (pack year)	<0.001	m: 1.016 (1.006; 1.025); w: 0.950 (0.895; 1.009)
		Healthy diet (1 = yes, 0 = no)	0.626	1.304 (0.757; 2.245)
		Physically active (1 = yes,0 = no)	0.235	1.542 (0.934; 2.544)
**B) Metabolic**				
	Early AMD^b^	Age (year)	<0.001	m: 1.055 (1.038; 1.072); w: 1.034 (1.019; 1.049)
		Sex (0 = men, 1 = women)	0.019	1.540 (1.089; 2.161)
		Smoking (pack year)	0.061	m: 1.007 (1.000; 1.015); w: 0.989 (0.972; 1.006)
		BMI (kg/m^2^)	0.563	1.009 (0.979; 1.040)
		T2DM (1 = yes, 0 = no)	0.640	1.151 (0.644; 2.056)
		Hypertension (1 = yes, 0 = no)	0.163	1.229 (0.921; 1.640)
		HDL-Cholesterol (mg/dl)	0.805	1.001 (0.993; 1.010)
		LDL-Cholesterol (mg/dl)	0.063	0.997 (0.994; 1.000)
	AREDS steps 4+	Age (year)	<0.001	m: 1.092 (1.058; 1.127); w: 1.090 (1.052; 1.129)
		Sex (0 = men, 1 = women)	0.017	1.232 (0.569; 2.665)
		Smoking (pack year)	0.004	m: 1.014 (1.004; 1.024); w: 0.950 (0.894; 1.009)
		BMI (kg/m^2^)	0.522	1.033 (0.978; 1.092)
		T2DM (1 = yes, 0 = no)	0.407	0.463 (0.135; 1.590)
		Hypertension (1 = yes, 0 = no)	0.875	1.144 (0.687; 1.905)
		HDL-Cholesterol (mg/dl)	0.980	1.002 (0.986; 1.018)
		LDL-Cholesterol (mg/dl)	0.210	0.994 (0.988; 1.001)

Shown are results for the association with **A)** lifestyle or **B)** metabolic factors. The outcome is early AMD versus controls and AREDS steps 4+ versus controls (270 or 270 subjects with early AMD for A or B, respectively, 72 or 71 subjects with AREDS steps 4+, 2,208 or 2,182 AMD-free subjects). These four logistic regression models include as covariates: age (modelled linearly), sex (0 = men, 1 = women), pack year (linearly), sex x age, sex x pack year. Results modelling age non-linearly were very similar (data not shown).

Abbreviations: CI = confidence interval; m = men, w = women; AREDS = Age-Related Eye Disease Study

^a^) P-value for association or, where applicable, Likelihood Ratio Test testing the main effect for age and interaction sex x age, or main effect smoking and interaction sex x smoking simultaneously.

^b^) Early AMD is defined as AREDS severity steps 2–9.

Since both parameters “developing AMD” and “deteriorating metabolic processes” are known aspects of aging, it is perceivable that AMD development and metabolic processes had common mechanisms and one could expect a coincidence of AMD and deteriorated metabolic factors. We thus analysed the association of BMI, T2DM, hypertension, HDL- and LDL-Cholesterol with early AMD. While subjects with early AMD features showed a tendency towards adverse health for all metabolic parameters (higher BMI, higher LDL-C, lower HDL-C, more T2DM, more hypertension, data not shown), this was due to confounding by age: no association was shown when adjusting for age linearly (**Table 4B**) or non-linearly (data not shown), nor when restricting to AREDS severity steps 4+ (**Table 4B**).

### Accounting for the trend of increased early AMD by age

As lifestyle factors and metabolic factors change with age, it is perceivable that some of the age-effects on early AMD features are explained by lifestyle (e.g. reduced physical activity) or metabolic factors (e.g. increased BMI, more T2DM or hypertension, deteriorated lipids). In our models with lifestyle or metabolic factors as covariates (as in **Tables 3 and 4**), we found that the increased risk of early AMD features by age did not change upon adjusting for lifestyle or metabolic factors (**S8 Table**). We found similar results when restricting to subjects graded as AREDS severity steps 4+ or steps 2+3 (**S8 Table**). This underscores that the age-trend of increased risk for early AMD features appears to be independent of age-related changes in lifestyle or metabolic processes.

## Discussion

### Summary of main findings

We provide prevalence estimates for early AMD features and—with noted caution due to small numbers—for late AMD for a general German adult population aged 24 to 75 years. This does not only fill an existing gap of data on AMD features in Germany [38], but also provides evidence for early AMD features in subjects younger than 50 years of age.

Besides extending AMD grading to younger adults, we are—to our knowledge—the first to establish a dose-response relationship of the risk for early AMD features by years of age and by pack years smoked during a lifetime in detail and in a sex-specific fashion. Our data establishes a linear trend of increased risk of early AMD features by age for men, which underscores that early AMD may not be a disease with ad hoc appearance at an age of 50, but bares a risk for the younger men just as well. For women, the relationship appears to be more complex with a linear increase for those younger than 40, a plateau for those aged 40 to 55 years, and a linearly increased risk for early AMD features thereafter. The observed age-effect is independent of lifestyle or metabolic factors. We also substantiate a dose-response relationship by pack year for men, but not for women. Our data did not support any association of AMD with other lifestyle or metabolic factors.

### Prevalence of early AMD features and late AMD in light of other studies

Quantifying the prevalence of early and late stage AMD is an important determinant for health care and research management. In our survey of the adult general population in Bavaria, we observed a frequency of 10.9% for early and 0.2% for late AMD, yielding a prevalence estimate of 11.4% for early and 0.2% for late AMD when standardized to the Bavarian population. While our definition of early AMD was based on the AMD severity steps 2–9 as recommended by the AREDS report no. 17 [33], there is a substantial debate on how to define early AMD.[26] Often, the term “intermediate AMD” is utilized describing the presence of large drusen (> 125μm) and/or any AMD-related pigmentary abnormalities (hyper- or hypopigmentation).[39] We found a standardized prevalence of 3.1% for pathologically more relevant signs of early AMD as defined by the AREDS severity steps 4–9.

A comparison of our AMD prevalence estimates with estimates from other studies in Germany is limited due to scarce data. Such estimates were completely lacking until recently (2014) when the Gutenberg Health Study [40] reported a prevalence of 0.2% for late AMD and 11.9% for early AMD. This is in line with our data, despite the fact that the Gutenberg Health Study has used a different grading scheme for defining early AMD.[40]

The different grading schemes are also the reason why a broader comparison to other studies is hampered. There had been no gold standard grading system, when we started the grading of our > 5,000 fundus images in 2013. We had chosen to apply the well-established AREDS 9-step severity scale [33], which is an extension of the Wisconsin Age-Related Maculopathy Grading System (WARMGS) [41, 42], for several reasons: (i) the AREDS 9-step severity scale does not rely on drusen morphology, which might be a more subjective criterion, but on a well demarcated and measurable drusen area; (ii) the 9 steps allow for a finely graduated analysis of early AMD features, the clinical relevance of which being underscored by our observed trend of increased severity steps by years of age (see **S6 Table**); (iii) the AREDS 9-step severity scale had been described to predict well the progression to late AMD, as shown by the AREDS group [33], and it had been validated in the Complications of Age-related Macular Degeneration Prevention Trial [43] (in fact very recently substantiated in the AREDS2 study [44]). While there had been also studies reporting early AMD progression prediction based on other grading systems [45], a direct comparison of progression prediction by each grading systems using a large longitudinal study had been (and still is) lacking.

In the meanwhile, later in 2013, Ferris et al. published a less complex classification system to be also used in routine clinical settings, focusing on main AMD features such as the presence or absence of large drusen and/or pigmentary abnormalities.[39] In 2014, the Three Continent AMD Consortium [26], an alliance of the Rotterdam Study, the Beaver Dam Eye Study, the Blue Mountain Eye Study and the Los Angeles Latino Eye Study, reported a sizable impact on early AMD prevalence estimates upon re-grading images of subgroups of their studies via a harmonized AMD grading system. This new severity scale developed by the Three Continent AMD Consortium integrated features of the WARMGS and the Rotterdam Study classification.[26] To give an idea of how our study data looks like for these two grading systems, we have re-graded a random subgroup of our data (10%) and yielded a raw early AMD frequency of 19.1% and 6.5% for the Clinical Classification and the Three Continent AMD Consortium severity scale, respectively (**S9 Table**). This would compare to 7.9% based on the AREDS steps 2–9 in this subgroup, which differs from our presented frequency of 10.9% in our total sample due to random fluctuation in the small subgroup. Still, this highlights differences between grading systems.

To our opinion it is still unclear which grading system to use as standard. Once an accepted standard for grading is agreed upon by the ophthalmological societies, re-grading of all population-based studies would enable a direct comparison. A current overview of published population-based studies, stating applied grading systems and definitions of early AMD, as well as corresponding prevalence estimates, is provided in **S10 Table**. There is a trend towards higher prevalence estimates from Northern Europe for both early and late AMD compared to Central European ancestry [46, 47], and a lower prevalence for early AMD in Asian studies [48, 49], as noted previously. Besides the acknowledged differences in the grading systems, differences might be due to different genetic background, climatic conditions, or lifestyle.

### Young adults with early AMD features might profit most from preventive measures

Despite of AMD being a disease in the elderly, we also find subjects with early AMD features based on colour fundus images in young adults under the age of 30 years. To our knowledge, our study is the first to describe early AMD features in subjects that young, but our data supports previous findings from the Gutenberg Health study reporting 3.8% of their participants with early AMD features in the youngest age group (35–44 years).[40] Several other population-based studies on AMD have limited their investigations to individuals older than 43 [26] or 55 years (see **S10 Table**) [1, 26, 47, 50, 51], which is the age where the appearance of AMD is expected.

There is a debate whether early and late AMD can be present in persons younger than 50 years of age and whether early AMD features found in young adults precede a clinically manifest late AMD.[25, 40] Our data showing a substantial proportion of early AMD features in the young allows for two different conclusions: (i) It can either imply that these AMD findings in young participants are truly AMD-related and contradict the current notion that AMD occurs only in subjects over 50 years of age. This would support a hypothesis of a common basis for juvenile and age-related macular degeneration. If these early AMD features in the young are truly preceding late AMD development, these young adults might profit the most from preventive measures, once such measures would be available. (ii) However, these early AMD lesions might also represent other non-AMD related pathologies mimicking features of AMD, or morphologies of a more static or less progressive nature. This could be the case particularly for minor early AMD lesions, as defined by AREDS severity steps 2+3. One argument underlining this caution is the progression rate to advanced AMD steps described by the AREDS group [33]: While 47% of individuals at AREDS steps 4+ progressed to step ≥7 or late AMD in 5 years, only 4% progressed among those with AREDS steps 2+3 at initial evaluation, compared to 1% among those with step 1.[33] This indicates that features of early AMD at AREDS steps 2+3 may not always equate to early AMD disease, as these persons are at lower risk compared to those with AREDS steps 4+, however showing some increased risk compared to those with AREDS step 1. As this progression data is on subjects with 55 to 80 years of age at baseline [33], the questions remains whether young subjects with AREDS steps 2+3 progress into clinically manifest AMD disease (steps ≥ 7 or late AMD) with the same velocity as elderly subjects in the same category. This would require large studies in the young with a long enough longitudinal observation to derive progression rates into clinically manifest AMD.

### Novel findings about the aging of the eye

There is a large debate on a dependency of AMD prevalence on sex with some studies reporting a higher prevalence of early AMD or NV AMD among women [20, 52], while most others reported no difference.[3, 13]. Our population-based data did not to indicate a difference in the prevalence of early AMD features between men and women; however, our numbers on late AMD were too low for drawing conclusions.

There is numerous work on the increased risk of early AMD features by age. However, only few–if any–evaluated whether or not the risk increase is linear by year of age. Generalized additive models allowing for a non—linear trend by age and an interaction between sex and age enabled us to detect a sex-differential trend of increased risk for early AMD features by age: a linearly increasing risk by years of age in men was contrasted by a non-linear relationship in women with an increased risk by year until the age of 40, a steady risk around 40 to 55 years of age, and a trend of increased risk by year of age thereafter. This could be explained by a cohort effect, since women aged 40 to 55 years at our survey were born between 1945 and 1960 and might have been imprinted by a lifestyle that was very different from those of men and from women of other birth cohorts. An alternative explanation could be hormonal changes in women of that age. Estrogen has been described as an anti-oxidative and anti-inflammatory capacity that may prevent the development of AMD, a protective effect that might diminish after menopause. [53] Longitudinal studies would be required to exclude a cohort effect.

Interestingly, the age-trend for both men and women was independent of lifestyle factors and metabolic parameters like blood pressure, fat, glucose, or lipid status. This supports the notion that lifestyle or the “aging” of metabolic processes does not explain the age-effect on early AMD features.

### Associations with smoking

Current smoking, ever smoking, or pack year smoked during a lifetime have been described as associated with early and late forms of AMD.[8–11, 13, 14] However, these associations were often statistically significant only in subgroups (men or women, early or late AMD) [12] and there are also studies reporting no association of smoking with early or late AMD, e.g. in a large Asian population.[54] Our data demonstrates early AMD features to be associated with pack year in men, but not in women, and even supports a linear dose-response per pack year. Our data on elderly smoking women might be too sparse for drawing conclusions, possibly due to a cohort effect of a time when smoking was not yet fashionable for women. Despite the many studies investigating smoking with AMD and the clear suspicion of adverse health effects of smoking in both, men and women, our as much as previous data still lack a robust quantification of early AMD risk by smoking.

Considering that participant are usually unaware of their sub-clinical early AMD, the co-incidence of smoking and early AMD can be considered to be genuine and not due to individuals changing lifestyle upon diagnosis. Some longitudinal studies with baseline assessment of smoking showed association of smoking with incident late AMD, but not incident early AMD.[55] Longitudinal data are warranted to substantiate the role of smoking for incident early AMD.

### Lack of association with other lifestyle or metabolic factors

A healthy lifestyle including physical activity or a healthy diet with a high proportion of fish and vegetable consumption is prone to be protective for numerous chronic diseases including AMD.[24] The KORA survey which adopts standardized questions on physical activity [30] or the 24-item food frequency questionnaire [31] depicts no association for these lifestyle factors. Clearly, assessment of these factors is utterly difficult, as it is restricted to self-report and considered to be subject to substantial measurement error.[56] Thus, a subtle association might have been missed.

Metabolic parameters like BMI, T2DM, hypertension, or lipid status have also been a target for epidemiological studies as potentially being associated with AMD due to the clear relationship of all of these factors with aging.[9, 20, 51] Moreover, cholesteryl ester transfer protein (CETP) and hepatic lipase (LIPC) provide a genetic link of lipids to AMD.[57] Our data with full control of potential confounding by age or smoking did not find an association or early AMD with any metabolic factor, not even for HDL-cholesterol, for which there have been several reports with inconclusive results.[13, 58, 59]

### Strengths and limitations of our study

A strength of our investigation is the general population design with a high response rate and an unbiased assessment of lifestyle and metabolic factors. Also, our investigation is strongly supported by a detailed modelling of the dose-response-relationship by years of age and pack year smoked during a lifetime.

The full KORA-S4 survey reported a response of 67% [29], which is very high for a general population study. Still, this response implies that one third of the general population are missed and the processes of this selection are difficult to pinpoint: the data might be enriched for subjects at a generally better health or for subjects who are sensitive to health issues due to personal or family experiences. While not all KORA-S4 participants were eligible for the fundus sub-study due to practical reasons (2,842 subjects eligible, 100%), we had little drop-out from non-participation to the fundus photography (99.9% agreed), un-successful imaging (96.8% imaged for each eye), or insufficient image quality or competing eye disease prohibiting grading (89.6% analysable subjects). The drop-out was, to some extent, related to higher age, due to smaller pupil size and thus deteriorated image quality prohibiting grading [60, 61]. Based on the data from the rest of the KORA survey, we can show that we have a tendency towards younger subjects and the corresponding metabolic make-up, but that the overall metabolic status or lifestyle behaviour in our fundus sub-study is comparable to the full KORA-S4 survey when adjusting for age.

Another strength of our AMD assessment is that we have restricted our analyses to subjects with both eyes gradable, while others have usually included subjects also when only one eye was gradable, focussing on the most severe eye if grading was possible for both eyes. By this, our data would not be subject to over-stating early AMD features.

A limitation of our data is its current lack of a longitudinal follow-up. We can thus only provide prevalence, but not incidence estimates, and can only assess association with smoking, but cannot establish smoking as a risk factor for the development of early AMD. More studies with longitudinal follow-up will be important to substantiate incidence and risk factors. Such studies will also be able to clarify whether the young subjects with early AMD features are at increased risk for late AMD. If that was true, these early AMD features in the young would be truly early AMD and the definition of AMD as being a disease purely in the elderly would need to be reconsidered.

### Conclusion and outlook

Our data fills a gap of prevalence estimates for early AMD features and late AMD in Germany and Central Europe to complement the few existing data sets available. Our data shows that early AMD features exist also in young adults and might be under-acknowledged. Besides confirming the solid association of AMD with age, we establish a sex-differential trend by years of age, and underscore an aging of the eye that is independent of lifestyle or metabolic processes. We can substantiate the role of smoking for early AMD features and a dose-response relationship, but only for men. Our data does not support a link between early AMD features with healthy diet, physical activity, nor with any metabolic factor, including HDL-Cholesterol.

## Supporting Information

S1 Fig Digitally designed and imported grid for AMD grading. (PDF)

S1 Table Grading of early AMD features and definition of late AMD in KORA-S4. (PDF)

S2 Table Exclusion of subjects in the KORA-S4 fundus sub-study. (PDF)

S3 Table Life style factors by 10-year age-groups separately for men and women. (PDF)

S4 Table General characteristics of the overall KORA-S4 participants compared to those analysed in this fundus sub-study. (PDF)

S2 Fig Age distribution of the subjects analysed in the fundus sub-study compared to the full KORA-S4 survey. (PDF)

S3 Fig AMD frequencies by sex and ten-year-age groups. (PDF)

S5 Table Relative risk estimates of early/late AMD by sex and ten-year age-groups. (PDF)

S6 Table Frequency of early AMD by severity steps and age-groups. (PDF)

S7 Table AMD grading per eye. (PDF)

S4 Fig The age-trend or risk modelled separately for AREDS severity steps 2+3 and steps 4+ in men and women. (PDF)

S5 Fig The trend of early AMD risk by pack year separately for men and women. (PDF)

S8 Table An accounting of the age-trend of early AMD risk. (PDF)

S9 Table Early AMD grading results comparing three grading systems. (PDF)

S10 Table AMD prevalence in our KORA-S4 fundus sub-study in the context of previously published population-based studies. (PDF)
