# Supplementary material for: Features of Age-Related Macular Degeneration in the General Adults and Their Dependency on Age, Sex, and Smoking: Results from the German KORA Study
Source: PLoS One. 2016 Nov 28;11(11):e0167181. doi: 10.1371/journal.pone.0167181 (PMC5125704; doi:10.1371/journal.pone.0167181)
Supplement: S1 Fig — (PDF) [file pone.0167181.s001.pdf]

**S1 Fig. Digitally designed and imported grid for AMD grading.**

**A)** The optic disc is used as scale reference (= left circle, adjusted for each image). Radii of the grid circles are one-third, 1, and 2 disc diameters, respectively, and their areas are 4/9, 4, and 16 disc areas (DAs). When the diameter of the optic disc is assumed to be 1500 μm, the radius of the central circle of the grid is 500 μm, that of the middle (inner) circle is 1500 μm, and that of the outer circle is 3000 μm. C (=”center”), I (=”inner”), O (=”outer”) resemble standard circles for determination of drusen area following AREDS report No 17.[1] **B)** Non-mydriatic colour fundus image of the central retina with digitally imported grid, adapted in size and centred on the fovea. Multiple Drusen and pigmentary abnormalities are visible within the grid area, resulting in AREDS severity step 4.

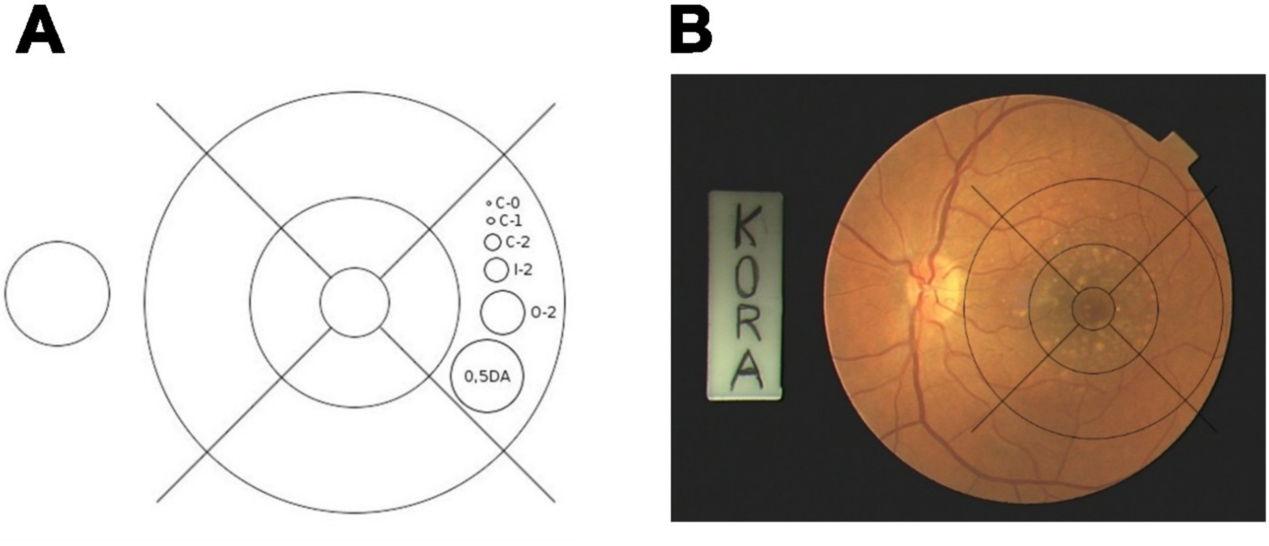

**References**

1. Davis MD, Gangnon RE, Lee LY, Hubbard LD, Klein BE, Klein R, et al. The Age-Related Eye Disease Study severity scale for age-related macular degeneration: AREDS Report No. 17. ArchOphthalmol. 2005;123(11):1484-98.
