# Supplementary material for: Features of Age-Related Macular Degeneration in the General Adults and Their Dependency on Age, Sex, and Smoking: Results from the German KORA Study
Source: PLoS One. 2016 Nov 28;11(11):e0167181. doi: 10.1371/journal.pone.0167181 (PMC5125704; doi:10.1371/journal.pone.0167181)
Supplement: S1 Table — (PDF) [file pone.0167181.s002.pdf]

S1 Table. Grading of early AMD features and definition of late AMD in KORA-S4.

Our grading of early AMD was based on the 9-step Age-Related Eye Disease Study severity scale for early AMD (AREDS report no. 17).[1] Allocation to steps was performed per eye. The eye with the more severe diagnosis was used to classify the person. GA is only defined as GA if no NV present, NV means that there can be GA additional in the same eye or in the other.

	AREDS severity step	Drusen area	Increased pigment		Depigmentation-GA
**No AMD**	**1**	<C-1	0		0
**Early AMD**	**2**	≥C-1, <C-2	0		0
		<C-1	≥Q	and/or	≥Q, <I-2
	**3**	≥C-2, <I-2	0		0
	**4**	≥I-2, <O-2	0		0
		≥C-1, <I-2	≥Q	and/or	≥Q, <I-2
		<C-2	≥0		≥I-2, <0.5 DA
	**5**	≥O-2, <0.5 DA	0		0
		≥I-2, <O-2	≥Q		≥Q, <I-2
		≥C-2, <I-2	≥0	and/or	≥I-2, <0.5 DA
	**6**	≥0,5 DA	0		0
		≥O-2, <0.5 DA	≥Q	and/or	≥Q, <I-2
		≥I-2, <O-2	≥0		≥I-2, <0.5 DA
	**7**	≥0.5 DA	≥Q	and/or	≥Q, <I-2
		≥O-2, <0.5 DA	≥0		≥I-2, <0.5 DA
	**8**	≥0,5 DA	≥Q		≥I-2, <0.5 DA
		Any	≥0		≥0.5 DA
	**9**	Any	≥0		Non-central GA
**Late AMD**		Any	≥0		Central GA
		Any	≥0		Scaring and/or neovascular AMD

This table is sorted by increased severity.
Abbreviations: GA = geographic atrophy; DA = disc area; 0 = not present; >0 = present; Q = questionable; C (=”center”), I (=”inner”), O (=”outer”), 1 or 2 = standard circles as defined by AREDS report no. 17 (also shown in S1 Fig A)[1];

References

1. Davis MD, Gangnon RE, Lee LY, Hubbard LD, Klein BE, Klein R, et al. The Age-Related Eye Disease Study severity scale for age-related macular degeneration: AREDS Report No. 17. ArchOphthalmol. 2005;123(11):1484-98.
