# Supplementary material for: Features of Age-Related Macular Degeneration in the General Adults and Their Dependency on Age, Sex, and Smoking: Results from the German KORA Study
Source: PLoS One. 2016 Nov 28;11(11):e0167181. doi: 10.1371/journal.pone.0167181 (PMC5125704; doi:10.1371/journal.pone.0167181)
Supplement: S2 Table — (PDF) [file pone.0167181.s003.pdf]

S2 Table. Exclusion of subjects in the KORA-S4 fundus sub-study.

Shown is the number of subjects after each exclusion step and the percentage compared to those eligible for the fundus sub-study.

Sample description	n (%)
KORA-S4 participants	4261
KORA-S4 participants eligible for fundus sub-study	2842 (100%)
participants agreeing to fundus sub-study	2840 (99.9%)
participants with at least one acquired fundus image for any eye (imaged subjects)	2789 (98.1%)
participants with at least one acquired fundus image for each eye	2751 (96.8%)
participants with at least one gradable fundus image for any eye	2613 (91.9%)
participants with at least one gradable fundus image for each eye (gradable subjects)	2569 (90.4%)
participants with gradable fundus image for each eye after exclusion of subjects with competing eye condition[a] (analysed subjects)	2546 (89.6%)

[a]) Competing eye conditions include obscuring lesions (e.g. cataract) and lesions that were considered to be the result of generalized retinal disease, such as diabetic retinopathy, high myopia, trauma, congenital diseases, or photocoagulation for reasons other than for choroidal neovascularization.
