# Supplementary material for: Features of Age-Related Macular Degeneration in the General Adults and Their Dependency on Age, Sex, and Smoking: Results from the German KORA Study
Source: PLoS One. 2016 Nov 28;11(11):e0167181. doi: 10.1371/journal.pone.0167181 (PMC5125704; doi:10.1371/journal.pone.0167181)
Supplement: S3 Table — (PDF) [file pone.0167181.s004.pdf]

S3 Table. Life style factors by 10-year age-groups separately for men and women.

Shown are the proportions of subjects smoking, those adopting a healthy diet or pertaining a physically active life style in the 2,546 analysed subjects.

Age groups (years)	# analysed subjects with the variable available	All	<30	30-39	40-49	50-59	60-69	70-75
# subjects in analysed sample		n=2546	n=229	n=644	n=548	n=517	n=452	n=156
**Men**								
Current smoker versus other	1275	32.8%	40.4%	49.4%	36.1%	28.7%	16.5%	10.3%
Ex-smoker versus other	1275	37.1%	20.2%	21.9%	35.3%	41.9%	52.4%	62.1%
Ever smoking vs. never	1275	69.9%	60.6%	71.3%	71.4%	70.6%	68.8%	72.4%
≥5 pack year versus 0	1045	65.2%	50.0%	64.5%	67.3%	67.9%	65.7%	67.1%
Physically active	1266	50.2%	66.1%	53.4%	54.0%	46.7%	44.7%	32.2%
Healthy diet	1266	48.4%	28.4%	40.6%	48.5%	47.8%	63.3%	64.4%
**Women**								
Current smoker versus other	1267	25.1%	9.4%	36.8%	24.8%	19.2%	8.2%	1.6%
Ex-smoker versus other	1267	26.0%	25.8%	22.3%	31.2%	28.7%	23.6%	21.7%
Ever smoking vs. never	1267	51.1%	50.8%	57.5%	59.2%	53.7%	35.5%	29.0%
≥5 pack year versus 0	1030	51.5%	33.3%	47.1%	50.0%	48.1%	27.6%	19.0%
Physically active	1262	52.0%	50.0%	56.5%	54.6%	46.5%	50.5%	47.1%
Healthy diet	1262	64.6%	53.3%	55.6%	58.2%	63.8%	80.5%	82.4%

Current smokers are defined as regular smokers, currently smoking ≥ 1 cigarette per day, and occasional smokers, currently smoking < 1 cigarette per day.; ever smokers are defined as current or ex-smokers; pack years are defined as number of packs (20 cigarettes per pack) smoked per day times the number of years of smoking; physically active is defined as ≥ 1 hour of activity per week during leisure time in summer and winter; healthy diet is defined as a healthy diet score above the median of the analysed sample (median score = 15.00);
