# Supplementary material for: Features of Age-Related Macular Degeneration in the General Adults and Their Dependency on Age, Sex, and Smoking: Results from the German KORA Study
Source: PLoS One. 2016 Nov 28;11(11):e0167181. doi: 10.1371/journal.pone.0167181 (PMC5125704; doi:10.1371/journal.pone.0167181)
Supplement: S4 Table — (PDF) [file pone.0167181.s005.pdf]

S4 Table. General characteristics of the overall KORA-S4 participants compared to those analysed in this fundus sub-study.

Shown are participant characteristics for the total study sample (n=4,261), for those subjects without any acquired fundus image (without image, n=1,472), for subjects without grading possible for each eye or with a competing retinal disease (n=243), and for those with gradable fundus image for each eye and no competing retinal disease (analysed subjects, n=2,546). Also shown is a P-value for difference between the analysed subjects and those subjects the un-gradable/with competing retinal disease and between the analysed and those without image computed as linear/logistic regression without and with adjusted for age and sex (where applicable).

	n	All	Without image	With image		P-value for difference			
				Not gradable / competing retinal disease	Analysed	Analysed vs. not gradable / competing retinal disease		Analysed vs. without image	
						Unadj.	Adj.	Unadj.	Adj.
Age [years], mean ± SD	4261	49.18 ± 13.94	50.26 ± 14.00	60.48 ± 11.41	47.48 ± 13.56	$9.80*10^{-46}$	$5.84*10^{-46}$	$7.56*10^{-10}$	$4.86*10^{-10}$
Age ≥ 50 years, n (%)	4261	2093 (49.12)	764 (51.90)	204 (83.95)	1125 (44.19)	$6.11*10^{-26}$	$4.21*10^{-26}$	$2.41*10^{-6}$	$1.69*10^{-6}$
Men, n (%)	4261	2090 (49.05)	700 (47.55)	112 (46.09)	1278 (50.20)	0.22	0.11	0.11	0.06
**Lifestyle factors**									
Current smoker[a], n (%)	4256	1107 (26.01)	325 (22.09)	46 (18.93)	736 (28.95)	$1.02*10^{-3}$	0.74	$2.19*10^{-6}$	$7.76*10^{-4}$
Ex-smoker, n (%)	4256	1332 (31.30)	438 (29.78)	91 (37.45)	803 (31.59)	0.06	0.96	$8.20*10^{-4}$	$1.39*10^{-3}$
Never smoker, n (%)	4256	1817 (42.70)	708 (48.13)	106 (43.62)	1003 (39.46)	0.21	0.99	$9.05*10^{-8}$	$4.73*10^{-6}$
Pack years[b], mean ± SD	4174	11.11 ± 18.37	10.45 ± 18.67	14.54 ± 21.71	11.16 ± 17.81	$5.79*10^{-3}$	0.30	0.24	0.068
Physically active[c], n (%)	4242	2064 (48.66)	666 (45.28)	107 (44.03)	1291 (51.07)	0.037	0.55	$4.14*10^{-4}$	$4.36*10^{-3}$
Healthy diet[d], n (%)	4241	2412 (56.87)	833 (56.63)	167 (69.01)	1412 (55.85)	$9.33*10^{-5}$	0.26	0.63	0.29
**Metabolic parameters**									
BMI [kg/m²][e], mean ± SD	4224	27.22 ± 4.73	27.63 ± 4.72	28.44 ± 4.33	26.87 ± 4.73	$6.98*10^{-7}$	0.34	$1.09*10^{-6}$	$1.28*10^{-3}$
T2DM[f], n (%)	4255	170 (4.00)	57 (3.88)	24 (9.88)	89 (3.50)	$4.33*10^{-6}$	0.07	0.54	0.62
Hypertension[g], n (%)	4243	1286 (30.31)	472 (32.11)	97 (40.08)	717 (28.33)	$1.43*10^{-4}$	0.76	0.01	0.35
HDL-C [mg/dl]	4214	57.71 ± 17.00	57.96 ± 17.10	57.20 ± 15.21	57.62 ± 17.12	0.71	0.41	0.54	0.95
LDL-C [mg/dl]	4209	137.47 ± 41.53	137.86 ± 39.84	149.86 ± 41.42	136.07 ± 42.29	$1.48*10^{-6}$	0.65	0.19	0.50

Abbreviations: SD = standard deviation; BMI = body-mass-index; T2DM = type 2 diabetes; HDL-C, LDL-C = high and low density lipoprotein cholesterol; unadj. = unadjusted; adj. = adjusted for age and sex if not indicated otherwise;
[a]) Current smokers are defined as regular smokers currently smoking ≥ 1 cigarette day, and occasional smokers, currently smoking < 1 cigarette per day.
[b]) Pack years are defined as number of packs (20 cigarettes per pack) smoked per day times the number of years of smoking.
[c]) Physically active is defined as ≥ 1 hour of activity per week during leisure time in summer and winter.
[d]) Healthy diet is defined as a healthy diet score above the median of the analysed sample (median score = 15.00).
[e]) BMI is defined as measured weight divided by squared measured body height.
[f]) T2DM is defined as a self-reported diagnosis or anti-diabetes medication intake.
[g]) Hypertension is defined as actually measured systolic blood pressure of ≥ 140 mmHg, diastolic blood pressure of ≥ 90 mmHg or corresponding medication taken, given that the participants were aware of having hypertension.
