# Supplementary material for: Features of Age-Related Macular Degeneration in the General Adults and Their Dependency on Age, Sex, and Smoking: Results from the German KORA Study
Source: PLoS One. 2016 Nov 28;11(11):e0167181. doi: 10.1371/journal.pone.0167181 (PMC5125704; doi:10.1371/journal.pone.0167181)

S2 Fig. Age distribution of the subjects analysed in the fundus sub-study compared to the full KORA-S4 survey.

Shown are the age distributions of the subjects in the full KORA-S4 survey (n=4,262), for the subjects analysed in this fundus sub-study (available and gradable fundus images for each eye, no competing retinal disease, n=2,546), and for the subjects not analysed (n=1,715). The age distribution for the analysed subjects is shifted towards the younger when compared to the full KORA-S4 survey.

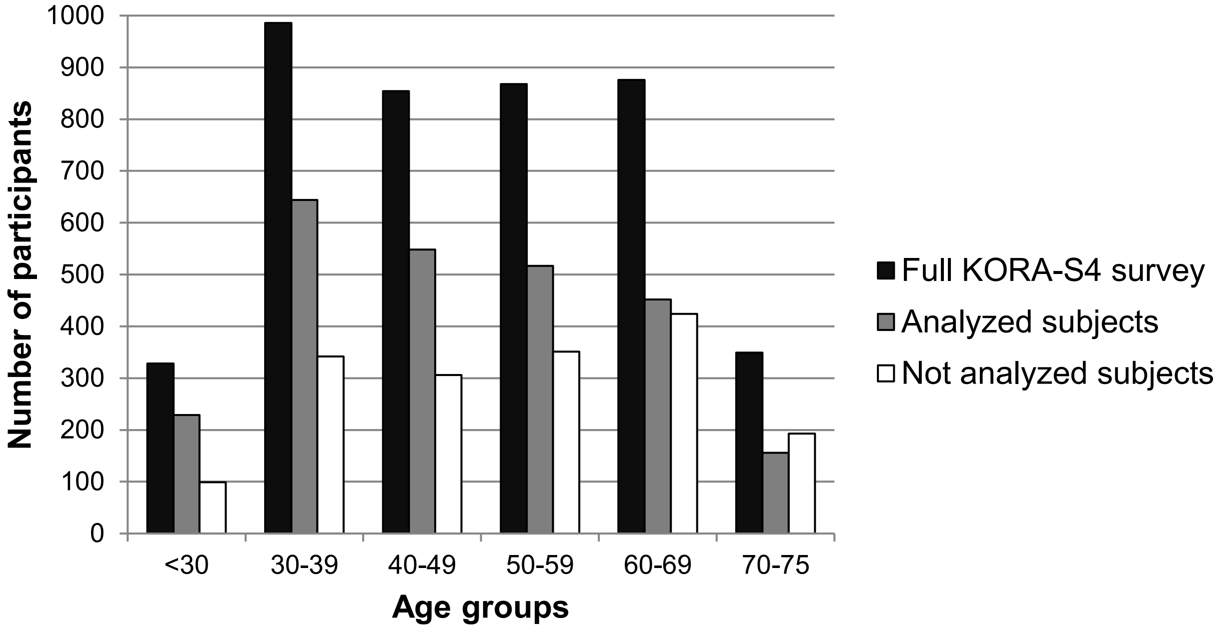

Supplement: S2 Fig — (PDF) [file pone.0167181.s006.pdf]
