# Supplementary material for: Features of Age-Related Macular Degeneration in the General Adults and Their Dependency on Age, Sex, and Smoking: Results from the German KORA Study
Source: PLoS One. 2016 Nov 28;11(11):e0167181. doi: 10.1371/journal.pone.0167181 (PMC5125704; doi:10.1371/journal.pone.0167181)

S3 Fig. AMD frequencies by sex and ten-year-age groups.

Shown is the proportion of subjects with no AMD, early AMD separated for AREDS severity steps 2+3 and steps 4+, or late AMD by 10-year age-groups, for both, men and women.

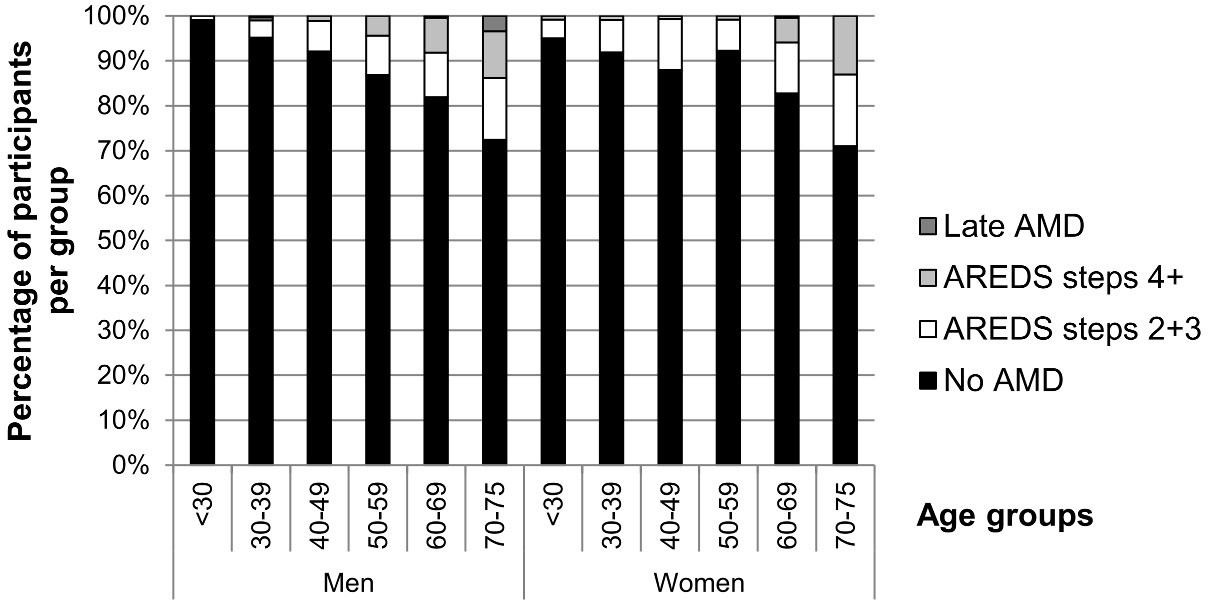

Supplement: S3 Fig — (PDF) [file pone.0167181.s007.pdf]
