# Supplementary material for: Features of Age-Related Macular Degeneration in the General Adults and Their Dependency on Age, Sex, and Smoking: Results from the German KORA Study
Source: PLoS One. 2016 Nov 28;11(11):e0167181. doi: 10.1371/journal.pone.0167181 (PMC5125704; doi:10.1371/journal.pone.0167181)
Supplement: S5 Table — (PDF) [file pone.0167181.s008.pdf]

S5 Table. Relative risk estimates of early/late AMD by sex and ten-year age-groups.

Shown are the relative risk estimates (with the odds ratio estimates in parentheses) in the 2,546 analysed subjects (age group 50-59 as reference).

Age groups (years)	<30	30-39	40-49	50-59	60-69	70-75
	n=229	n=644	n=548	n=517	n=452	n=156
**Overall**						
Early AMD[a]	0.29 (0.26)	0.60 (0.57)	0.94 (0.94)	Reference	1.62 (1.76)	2.47 (3.08)
AREDS steps 2+3	0.33 (0.30)	0.70 (0.67)	1.15 (1.14)	Reference	1.34 (1.45)	1.86 (2.31)
AREDS steps 4+	0.16 (0.15)	0.29 (0.27)	0.34 (0.33)	Reference	2.45 (2.66)	4.26 (5.30)
Late AMD (GA and/or NV)	Reference	Reference	Reference	Reference	Reference	15.32 (19.21)
**Men**						
Early AMD[a]	0.07 (0.06)	0.34 (0.31)	0.60 (0.56)	Reference	1.34 (1.42)	1.83 (2.19)
AREDS steps 2+3	0.10 (0.09)	0.44 (0.40)	0.77 (0.73)	Reference	1.13 (1.20)	1.57 (1.88)
AREDS steps 4+	-	0.15 (0.13)	0.26 (0.24)	Reference	1.77 (1.87)	2.35 (2.82)
Late AMD (GA and/or NV)	Reference	Reference	Reference	Reference	Reference	20.53 (25.62)
**Women**						
Early AMD[a]	0.64 (0.62)	1.04 (1.04)	1.55 (1.62)	Reference	2.16 (2.41)	3.72 (4.83)
AREDS steps 2+3	0.60 (0.58)	1.03 (1.04)	1.63 (1.71)	Reference	1.63 (1.82)	2.29 (2.97)
AREDS steps 4+	1.02 (0.99)	1.10 (1.10)	0.87 (0.91)	Reference	6.65 (7.42)	15.91 (20.66)
Late AMD (GA and/or NV)	Reference	Reference	Reference	Reference	Reference	n. c.

Abbreviations: AREDS = Age-Related Eye Disease Study; GA = geographic atrophy; NV = neovascularisation; n. c = not calculable due to zero cases in age group;
[a]) Early AMD is defined as AREDS severity steps 2-9.
