# Supplementary material for: Features of Age-Related Macular Degeneration in the General Adults and Their Dependency on Age, Sex, and Smoking: Results from the German KORA Study
Source: PLoS One. 2016 Nov 28;11(11):e0167181. doi: 10.1371/journal.pone.0167181 (PMC5125704; doi:10.1371/journal.pone.0167181)
Supplement: S6 Table — (PDF) [file pone.0167181.s009.pdf]

S6 Table. Frequency of early AMD by severity steps and age-groups.

Shown are the proportion of subjects with AREDS severity steps 1-9 and late AMD in the KORA-S4 fundus sub-study with gradable fundus images for each eye (n=2,546). Shown are the numbers and percentages of subjects in age-groups.

**Age groups (years)**	**<30**	**30-39**	**40-49**	**50-59**	**60-69**	**70-75**	**Total**, n (%)
**Participants**, n (%)	229 (8.99)	644 (25.29)	548 (21.52)	517 (20.31)	452 (17.75)	156 (6.13)	2546 (100)
**No AMD** (AREDS severity step 1), n (%)	222 (96.94)	602 (93.48)	493 (89.96)	462 (89.36)	372 (82.30)	112 (71.79)	2263 (88.88)
AREDS step 2, n (%)	6 (2.62)	24 (3.73)	40 (7.30)	27 (5.22)	32 (7.08)	16 (10.26)	145 (5.70)
AREDS step 3, n (%)	0 (0)	12 (1.86)	10 (1.82)	14 (2.71)	16 (3.54)	7 (4.49)	59 (2.32)
**AREDS severity steps 2+3**, n (%)	6 (2.62)	36 (5.59)	50 (9.12)	41 (7.93)	48 (10.62)	23 (14.74)	204 (8.01)
AREDS step 4, n (%)	1 (0.44)	5 (0.78)	4 (0.73)	9 (1.74)	19 (4.20)	8 (5.13)	46 (1.81)
AREDS step 5, n (%)	0 (0)	0 (0)	1 (0.18)	3 (0.58)	7 (1.55)	7 (4.49)	18 (0.71)
AREDS step 6, n (%)	0 (0)	0 (0)	0 (0)	2 (0.39)	2 (0.44)	3 (1.92)	7 (0.27)
AREDS step 7, n (%)	0 (0)	0 (0)	0 (0)	0 (0)	1 (0.22)	0 (0)	1 (0.04)
AREDS step 8, n (%)	0 (0)	0 (0)	0 (0)	0 (0)	0 (0)	0 (0)	0 (0)
AREDS step 9, n (%)	0 (0)	0 (0)	0 (0)	0 (0)	1 (0.22)	0 (0)	1 (0.04)
**AREDS severity steps 4+**, n (%)	1 (0.44)	5 (0.78)	5 (0.91)	14 (2.71)	30 (6.64)	18 (11.54)	73 (2.87)
**Late AMD** (GA and/or NV), n (%)	0 (0)	1 (0.16)	0 (0)	0 (0)	2 (0.44)	3 (1.92)	6 (0.24)

Abbreviations: AREDS = Age-Related Eye Disease Study; GA = geographic atrophy; NV = neovascularisation;
