# Supplementary material for: Features of Age-Related Macular Degeneration in the General Adults and Their Dependency on Age, Sex, and Smoking: Results from the German KORA Study
Source: PLoS One. 2016 Nov 28;11(11):e0167181. doi: 10.1371/journal.pone.0167181 (PMC5125704; doi:10.1371/journal.pone.0167181)
Supplement: S7 Table — (PDF) [file pone.0167181.s010.pdf]

S7 Table. AMD grading per eye.

Shown are number of subjects by eye-specific AMD grading comparing the right and left eyes in the 2,546 analysed subjects. A total of 2,380 participants revealed the same AMD status in both eyes.

		Left eyes			Total
		**No AMD** (AREDS step 1)	**Early AMD** (AREDS steps 2-9)	**Late AMD** (GA and/or NV)	
**Right eyes**	**No AMD** (AREDS step 1)	2263	82	0	2345
	**Early AMD** (AREDS steps 2-9)	78	117	2	197
	**Late AMD** (GA and/or NV)	2	2	0	4
	Total	2343	201	2	2546

Abbreviations: AREDS = Age-Related Eye Disease Study; GA = geographic atrophy; NV = neovascularisation;
Numbers of participants are given.
