# Supplementary material for: Features of Age-Related Macular Degeneration in the General Adults and Their Dependency on Age, Sex, and Smoking: Results from the German KORA Study
Source: PLoS One. 2016 Nov 28;11(11):e0167181. doi: 10.1371/journal.pone.0167181 (PMC5125704; doi:10.1371/journal.pone.0167181)

S4 Fig. The age-trend or risk modelled separately for AREDS severity steps 2+3 and steps 4+ in men and women.

Shown is the modeled trend of AMD risk (as log odds ratio) by years of age utilizing a thin plate regression spline for **A)** AREDS severits steps 2+3 risk in men (n cases = 90, n controls = 1,139), **B)** AREDS severits steps 2+3 risk in women (n cases = 114, n controls = 1,124), **C)** AREDS severits steps 4+ risk in men (n cases = 44, n controls = 1,139), and **D)** AREDS severits steps 4+ risk in women (n cases = 29, n controls = 1,124). Relative risk is given as log odds ratio with persons at the age of 50 as reference and 95% confidence interval shaded in grey. These relationships are derived by modeling the respective outcome with sex as covariate and a sex-specific thin plate regression spline for age.

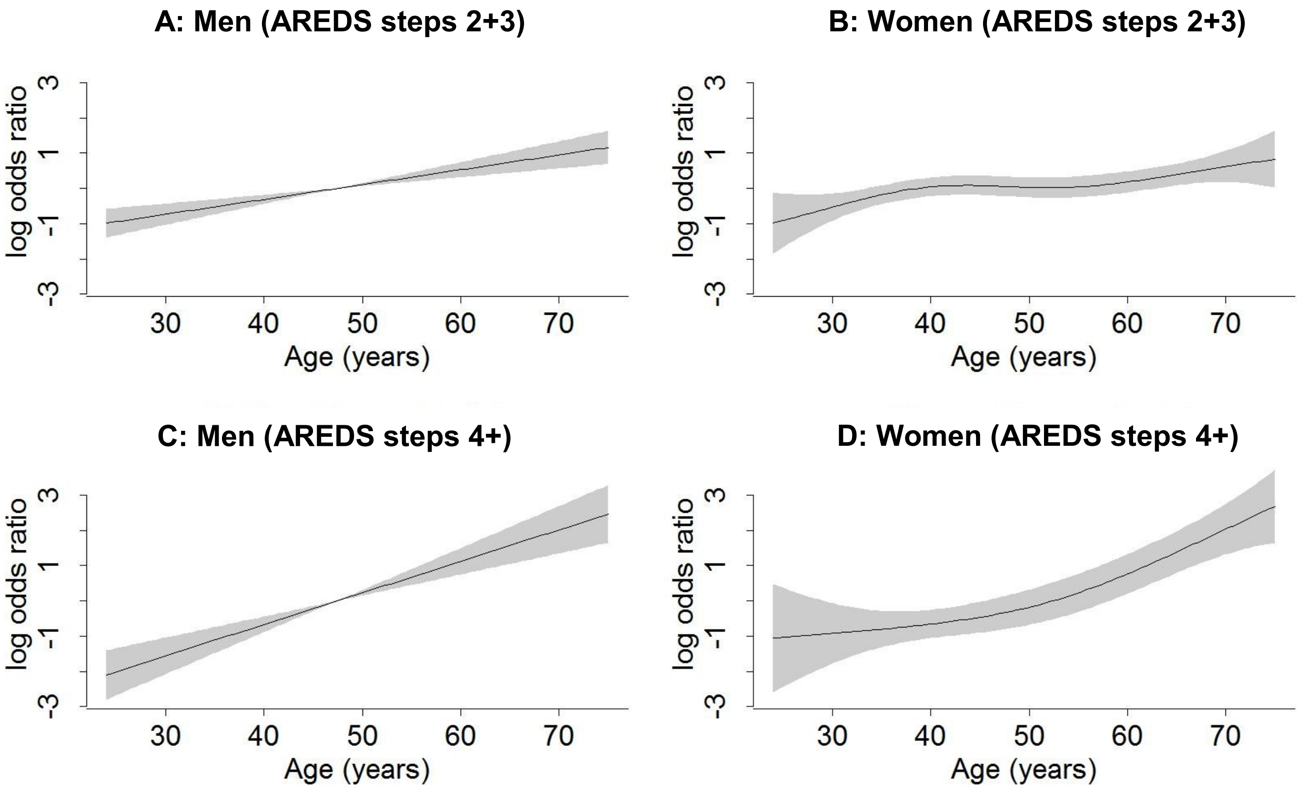

Supplement: S4 Fig — (PDF) [file pone.0167181.s011.pdf]
