# Supplementary material for: Features of Age-Related Macular Degeneration in the General Adults and Their Dependency on Age, Sex, and Smoking: Results from the German KORA Study
Source: PLoS One. 2016 Nov 28;11(11):e0167181. doi: 10.1371/journal.pone.0167181 (PMC5125704; doi:10.1371/journal.pone.0167181)

S5 Fig. The trend of early AMD risk by pack year separately for men and women.

Shown is the early AMD risk (on log scale) by pack year modelled by a sex-specific thin plate regression spline with the additional covariates sex, age (also modelled by a sex-specific thin plate regression spline) for **A)** men and **B)** women. The outcome is early AMD compared to controls (men: n=131 with early AMD, n=1,116 AMD-free; women: n=141 with early AMD, n=1,105 AMD-free).

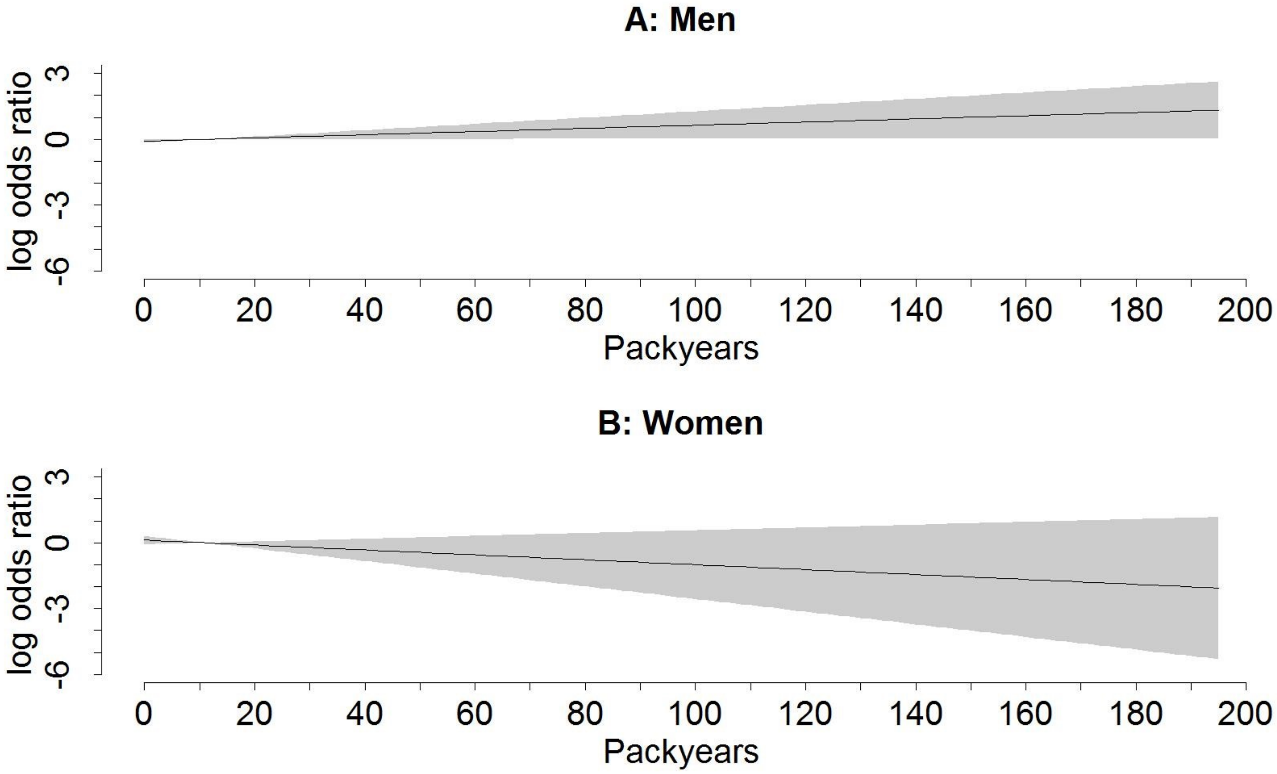

Supplement: S5 Fig — (PDF) [file pone.0167181.s012.pdf]
