# Supplementary material for: Features of Age-Related Macular Degeneration in the General Adults and Their Dependency on Age, Sex, and Smoking: Results from the German KORA Study
Source: PLoS One. 2016 Nov 28;11(11):e0167181. doi: 10.1371/journal.pone.0167181 (PMC5125704; doi:10.1371/journal.pone.0167181)
Supplement: S8 Table — (PDF) [file pone.0167181.s013.pdf]

S8 Table. An accounting of the age-trend of early AMD risk.

Shown are odds ratios (OR) per year and P-values from logistic regression models. All models include sex, age (linear or non-linear), and an interaction sex x age. The models adding PY also include an interaction sex x PY. The life style factors are healthy diet and physical activity; the metabolic factors are BMI, T2D, hypertension, HDL- and LDL-cholesterol (same models as in **Tables 3 and 4**). The outcome is early AMD versus AMD-free, AREDS severity steps 4+ versus AMD-free, or AREDS severity steps 2+3 versus AMD-free.

Model	Age linear			Age non-linear[b]	
	OR men (P)	OR women (P)	P sex difference[a]	df men	df women
**Early AMD[c]**					
age, sex	1.057 (<0.001)	1.035 (<0.001)	0.0344	1.001	3.283
+ PY	1.057 (<0.001)	1.033 (<0.001)	0.0281	1.000	3.272
+ PY, life style factors	1.056 (<0.001)	1.032 (<0.001)	0.0242	1.000	3.276
+ PY, metabolic factors	1.055 (<0.001)	1.034 (<0.001)	0.0606	1.000	3.228
**AREDS steps 4+**					
age, sex	1.093 (<0.001)	1.090 (<0.001)	0.8931	1.001	1.975
+ PY	1.093 (<0.001)	1.085 (<0.001)	0.7507	1.001	1.796
+ PY, life style factors	1.093 (<0.001)	1.084 (<0.001)	0.7156	1.001	1.811
+ PY, metabolic factors	1.092 (<0.001)	1.090 (<0.001)	0.9416	1.001	1.906
**AREDS steps 2+3**					
age, sex	1.043 (<0.001)	1.023 (0.002)	0.0929	1.003	2.917
+ PY	1.044 (<0.001)	1.022 (0.003)	0.0589	1.002	2.831
+ PY, life style factors	1.043 (<0.001)	1.020 (0.008)	0.0543	1.002	2.804
+ PY, metabolic factors	1.041 (<0.001)	1.021 (0.010)	0.1053	1.002	2.750

Abbreviations: PY = pack year; df = degrees of freedom with df ~ 1 indicating a linear relationship; AREDS = Age-Related Eye Disease Study;
[a]) P-value for the interaction of age and sex.
[b]) Using a thin plate regression spline.
[c]) Early AMD is defined as AREDS severity steps 2-9.
