# Supplementary material for: Features of Age-Related Macular Degeneration in the General Adults and Their Dependency on Age, Sex, and Smoking: Results from the German KORA Study
Source: PLoS One. 2016 Nov 28;11(11):e0167181. doi: 10.1371/journal.pone.0167181 (PMC5125704; doi:10.1371/journal.pone.0167181)
Supplement: S9 Table — (PDF) [file pone.0167181.s014.pdf]

S9 Table. Early AMD grading results comparing three grading systems.

Shown are the number of subjects of 10% of the KORA study participants (n=278) in a pairwise comparison by early AMD status using three grading systems: the AREDS 9-step severity scale [1], the Clinical Classification [2], and the Three Continent AMD Consortium severity scale [3]. **A**) Comparing the AREDS 9-step severity scale to the Three Continent AMD Consortium severity scale. **B**) Comparing the AREDS 9-step severity scale to the Clinical Classification. **C**) Comparing the Three Continent AMD Consortium severity scale to the Clinical Classification.

**A**		**Three Continent AMD Consortium severity scale**		Total
		No AMD	Early AMD[a]	
**AREDS 9-step severity scale**	No AMD (AREDS step 1)	253	3	256
	Early AMD (AREDS steps 2-9)	7	15	22
Total		260	18	278

Abbreviations: AREDS = Age-Related Eye Disease Study;
[a]) Early AMD is defined as "mild early"+moderate early"+"severe early" AMD following guidelines.[3]

**B**		**Clinical Classification**		Total
		No AMD[a]	Early AMD[b]	
**AREDS 9-step severity scale**	No AMD (AREDS step 1)	220	36	256
	Early AMD (AREDS steps 2-9)	5	17	22
Total		225	53	278

Abbreviations: AREDS = Age-Related Eye Disease Study;
[a]) No AMD is defined as "no apparent aging changes"+"normal aging changes" following guidelines.[2]
[b]) Early AMD is defined as "early"+"intermediate" AMD according to guidelines.[2]

**C**		**Clinical Classification**		Total
		No AMD[a]	Early AMD[b]	
**Three Continent AMD Consortium severity scale**	No AMD	225	35	260
	Early AMD[c]	0	18	18
Total		225	53	278

[a]) No AMD is defined as "no apparent aging changes"+"normal aging changes" following guidelines.[2]
[b]) Early AMD is defined as "early"+"intermediate" AMD according to guidelines.[2]
[c]) Early AMD is defined as "mild early"+moderate early"+"severe early" AMD following guidelines.[3]

References

1. Davis MD, Gangnon RE, Lee LY, Hubbard LD, Klein BE, Klein R, et al. The Age-Related Eye Disease Study severity scale for age-related macular degeneration: AREDS Report No. 17. ArchOphthalmol. 2005;123(11):1484-98.

2. Ferris FL, III, Wilkinson CP, Bird A, Chakravarthy U, Chew E, Csaky K, et al. Clinical classification of age-related macular degeneration. Ophthalmology. 2013;120(4):844-51. doi: S0161-6420(12)01055-X [pii];10.1016/j.ophtha.2012.10.036 [doi].

3. Klein R, Meuer SM, Myers CE, Buitendijk GH, Rochtchina E, Choudhury F, et al. Harmonizing the classification of age-related macular degeneration in the three-continent AMD consortium. Ophthalmic Epidemiol. 2014;21(1):14-23. doi: 10.3109/09286586.2013.867512 [doi].
