# Supplementary material for: Features of Age-Related Macular Degeneration in the General Adults and Their Dependency on Age, Sex, and Smoking: Results from the German KORA Study
Source: PLoS One. 2016 Nov 28;11(11):e0167181. doi: 10.1371/journal.pone.0167181 (PMC5125704; doi:10.1371/journal.pone.0167181)
Supplement: S10 Table — (PDF) [file pone.0167181.s015.pdf]

S10 Table. AMD prevalence in our KORA-S4 fundus sub-study in the context of previously published population-based studies.

Shown are prevalence estimates from population-based cross-sectional studies of European ancestry sorted by number of participants. For comparison to non-European ancestry, meta-analysis data from Asia including 9 studies from 4 Asian populations [1] is added.

**Study name** (Location)	**# subjects**	**Age [years]**	**Grading scheme for early AMD**	**Definition of early AMD**	**Early AMD [%]**	**Late AMD (GA/NV) [%]**	**References**
**Meta-analysis Asia**	10919	40-79	WARMGS [2] or International Classification and Grading System for AMD [3]	Soft indistinct or reticular drusen; or any drusen type except hard indistinct drusen with pigmentary abnormalities or Soft drusen ≥63 µm, hyperpigmentation and/or hypopigmentation of the retinal pigment epithelium (RPE)	6.8	0.6	Kawasaki et al., 2010 [1]
**Rotterdam Study (RS)**[a] (Netherlands)							
Before harmonization	6251[b]	55-98	Rotterdam Study classification [4]	Soft, distinct (≥63 µm) or indistinct (≥125 µm) or reticular drusen with/without pigmentary abnormalities	7.6	1.6	Vingerling et al., 1995 [5] Klein et al., 2014 [6]
After harmonization	6251[b]	55-98	Three Continent AMD Consortium severity scale [6]	Small - intermediate drusen (<125 µm) with pigmentary abnormalities; or large drusen (≥125 µm) with/without drusen area ≥ 331.820 µm^2 with/without pigmentary abnormalities	17.4	1.6	Klein et al., 2014 [6]
**Age, Gene/Environment Susceptibility (AGES) Reykjavik Study** (Iceland)	5272[b]	≥66	Modified after WARMGS [2]	Soft drusen (distinct or indistinct, ≥ 63–300 µm) with pigmentary abnormalities; or large soft drusen (≥125 µm) with a large drusen area (>500 µm diameter-circle); or large soft instinct drusen(≥125 µm)	21.3	5.3	Jonasson et al., 2011 [7]

S10 Table continued

**Beaver Dam Eye Study (BDES)[a]** (USA)							
Before harmonization	4771[b]	43-86	WARMGS [2]	Soft indistinct or reticular drusen; or any drusen type except hard indistinct drusen with pigmentary abnormalities	18.7	1.8	Klein et al., 1992 [8]<br>Klein et al., 2014 [6]
After harmonization	4771[b]	43-86	Three Continent AMD Consortium severity scale [6]	Small - intermediate drusen (<125 μm) with pigmentary abnormalities; or large drusen (≥125 μm) with/without drusen area ≥ 331.820 $μm^2$ with/without pigmentary abnormalities	20.3	1.8	Klein et al., 2014 [6]
**European Eye Study (EUREYE)** (Norway, Estonia, Northern Ireland, France, Italy, Greece, Spain)	4753[b]	≥65	Rotterdam Study classification [4]	Soft, distinct (≥63 μm) or indistinct (≥125 μm) or reticular drusen with/without pigmentary abnormalities	15.4	3.3	Augood et al., 2006 [9]
**The Irish Longitudinal study on Ageing (TILDA)** (Republic of Ireland)	4751[b]	≥ 50	Modified after the International Classification and Grading System for AMD [3]	>10 hard drusen (<63 μm) and/or presence of soft drusen (>125 μm)	6.1	0.4	Akuffo et al., 2015 [10]
**Gutenberg Health Study (GHS)** (Mainz, Germany)	4340[b]	35–74	Modified after the Rotterdam Study classification [4]	Soft, distinct (≥63 μm) or indistinct (≥125 μm) or reticular drusen with/without pigmentary abnormalities	11.9	0.2	Korb et al., 2014 [11]
**Blue Mountains Eye Study (BMES)[a]** (Australia)							
Before harmonization	3583[b]	≥49	WARMGS [2]	Soft indistinct or reticular drusen; or soft distinct drusen with pigmentary abnormalities	4.0	1.8	Mitchell et al., 1995 [12]<br>Klein et al., 2014 [6]
After harmonization	3583[b]	≥49	Three Continent AMD Consortium severity scale [6]	Small - intermediate drusen (<125 μm) with pigmentary abnormalities; or large drusen (≥125 μm) with/without drusen area ≥ 331.820 $μm^2$ with/without pigmentary abnormalities	12.8	1.8	Klein et al., 2014 [6]

S10 Table continued

**Tromsø Eye Study (TES)** (Norway)	2631[b]	65–87	Modified after the International Classification and Grading System for AMD [3]	Large drusen >125 µm	24.1	3.5	Erke et al., 2014 [13]
**KORA-S4 fundus sub-study** (Augsburg, Germany)	2546[c]	25-74	AREDS 9-step severity scale [14]	AREDS Severity Steps 2-9 (drusen area ≥ C-1 with/without pigmentary abnormalities	11.4	0.2	Present manuscript

Abbreviations: WARMGS = Wisconsin age-related maculopathy grading system; AREDS = Age-Related Eye Disease Study; C-1 = central standard circle as defined by AREDS report no. 17 [14] (also shown in S1 Fig A);

[a]) Prevalence is adjusted for age and sex.

[b]) Images were acquired for each eye with at least one eye being gradable, the more severe eye was used to classify the person.

[c]) Images were acquired for each eye with each eye being gradable, the more severe eye was used to classify the person.

References

1. Kawasaki R, Yasuda M, Song SJ, Chen SJ, Jonas JB, Wang JJ, et al. The prevalence of age-related macular degeneration in Asians: a systematic review and meta-analysis. Ophthalmology. 2010;117(5):921-7. doi: 10.1016/j.ophtha.2009.10.007. PubMed PMID: 20110127.

2. Klein R, Davis MD, Magli YL, Segal P, Klein BE, Hubbard L. The Wisconsin age-related maculopathy grading system. Ophthalmology. 1991;98(7):1128-34. PubMed PMID: 1843453.

3. Bird AC, Bressler NM, Bressler SB, Chisholm IH, Coscas G, Davis MD, et al. An international classification and grading system for age-related maculopathy and age-related macular degeneration. The International ARM Epidemiological Study Group. Survey of ophthalmology. 1995;39(5):367-74. PubMed PMID: 7604360.

4. Klaver CC, Assink JJ, van Leeuwen R, Wolfs RC, Vingerling JR, Stijnen T, et al. Incidence and progression rates of age-related maculopathy: the Rotterdam Study. Investigative ophthalmology & visual science. 2001;42(10):2237-41. PubMed PMID: 11527936.

5. Vingerling JR, Dielemans I, Hofman A, Grobbee DE, Hijmering M, Kramer CF, et al. The prevalence of age-related maculopathy in the Rotterdam Study. Ophthalmology. 1995;102(2):205-10. PubMed PMID: 7862408.

6. Klein R, Meuer SM, Myers CE, Buitendijk GH, Rochtchina E, Choudhury F, et al. Harmonizing the classification of age-related macular degeneration in the three-continent AMD consortium. Ophthalmic Epidemiol. 2014;21(1):14-23. doi: 10.3109/09286586.2013.867512 [doi].

7. Jonasson F, Arnarsson A, Eiriksdottir G, Harris TB, Launer LJ, Meuer SM, et al. Prevalence of age-related macular degeneration in old persons: Age, Gene/environment Susceptibility Reykjavik Study. Ophthalmology. 2011;118(5):825-30. doi: S0161-6420(10)00931-0 [pii];10.1016/j.ophtha.2010.08.044 [doi].

8. Klein R, Klein BE, Linton KL. Prevalence of age-related maculopathy. The Beaver Dam Eye Study. Ophthalmology. 1992;99(6):933-43. PubMed PMID: 1630784.

9. Augood CA, Vingerling JR, de Jong PT, Chakravarthy U, Seland J, Soubrane G, et al. Prevalence of age-related maculopathy in older Europeans: the European Eye Study (EUREYE). ArchOphthalmol. 2006;124(4):529-35.

10. Akuffo KO, Nolan J, Stack J, Moran R, Feeney J, Kenny RA, et al. Prevalence of age-related macular degeneration in the Republic of Ireland. The British journal of ophthalmology. 2015;99(8):1037-44. doi: 10.1136/bjophthalmol-2014-305768. PubMed PMID: 25712825; PubMed Central PMCID: PMC4518752.

11. Korb CA, Kottler UB, Wolfram C, Hoehn R, Schulz A, Zwiener I, et al. Prevalence of age-related macular degeneration in a large European cohort: Results from the population-based Gutenberg Health Study. Graefes ArchClinExpOphthalmol. 2014.

12. Mitchell P, Smith W, Attebo K, Wang JJ. Prevalence of age-related maculopathy in Australia. The Blue Mountains Eye Study. Ophthalmology. 1995;102(10):1450-60. PubMed PMID: 9097791.

13. Erke MG, Bertelsen G, Peto T, Sjolie AK, Lindekleiv H, Njolstad I. Cardiovascular risk factors associated with age-related macular degeneration: the Tromso Study. Acta ophthalmologica. 2014;92(7):662-9. doi: 10.1111/aos.12346. PubMed PMID: 24460653.

14. Davis MD, Gangnon RE, Lee LY, Hubbard LD, Klein BE, Klein R, et al. The Age-Related Eye Disease Study severity scale for age-related macular degeneration: AREDS Report No. 17. ArchOphthalmol. 2005;123(11):1484-98.
